# Bradykinin Metabolism and Drug-Induced Angioedema

**DOI:** 10.3390/ijms241411649

**Published:** 2023-07-19

**Authors:** Sylwia Smolinska, Darío Antolín-Amérigo, Florin-Dan Popescu

**Affiliations:** 1 Department of Clinical Immunology, Wroclaw Medical University, 50-368 Wroclaw, Poland; 2 Servicio de Alergia, Hospital Universitario Ramón y Cajal, Instituto Ramón y Cajal de Investigación Sanitaria (IRYCIS), 28034 Madrid, Spain; dario.antolin@gmail.com; 3 Department of Allergology “Nicolae Malaxa” Clinical Hospital, “Carol Davila” University of Medicine and Pharmacy, 022441 Bucharest, Romania; florindanpopescu@gmail.com

**Keywords:** bradykinin metabolism, drug-induced angioedema

## Abstract

Bradykinin (BK) metabolism and its receptors play a central role in drug-induced angioedema (AE) without urticaria through increased vascular permeability. Many cardiovascular and diabetic drugs may cause BK-mediated AE. Angiotensin-converting enzyme inhibitors (ACEIs) and neprilysin inhibitors impair BK catabolism. Dipeptidyl peptidase-IV (DPP-IV) inhibitors reduce the breakdown of BK and substance P (SP). Moreover, angiotensin receptor blockers, thrombolytic agents, and statins may also induce BK-mediated AE. Understanding pathophysiological mechanisms is crucial for preventing and treating drug-induced AE.

## 1. Introduction

Angioedema (AE) is a transient subcutaneous or submucosal swelling that is non-pitting when pressure is applied. It differs from edema and is caused by an accumulation of fluid in the interstitium and characterized by persistent pitting with pressure. AE is a presenting manifestation that results from an underlying pathophysiologic process involving the localized or systemic release of vasoactive mediators, most frequently histamine or bradykinin (BK) [1,2]. Differentiating between histaminergic AE and BK-mediated AE is of utmost importance, not only due to the different treatment approaches but also because the latter can be more often life-threatening. Acute episodes of AE without accompanying urticaria characterize BK-mediated AE, and these do not respond to conventional treatment with systemic H_1_ antihistamines, corticosteroids, or adrenaline. BK-mediated AE may be hereditary, acquired, or drug-induced [1,3,4,5].

A review on BK metabolism and drug-induced AE is crucial for clinicians unfamiliar with such potentially severe BK-mediated adverse drug reactions, as it enhances their awareness, improves diagnosis, guides drug selection and monitoring, facilitates risk communication with patients, and enables important preventive measures. The rationale behind such a review is to equip clinicians with accurate knowledge so that patient safety can be enhanced and such potentially life-threatening drug reactions can be better understood, prevented, and managed. There is a great need to effectively communicate the AE risks associated with certain medications to patients. Being knowledgeable about drug-induced AE and its underlying mechanisms enables clinicians to provide clear and accurate information to patients, facilitating shared decision-making and informed consent.

## 2. Overview on Epidemiology of BK-Mediated AE

The overall incidence of AE induced by angiotensin-converting enzyme inhibitors (ACEI-AE), the most important cause of BK-mediated AE, is less than 1%. Still, this therapeutic class is the leading cause of drug-induced AE, accounting for up to 40% of emergency room visits for AE, with relatively constant and persistent annual risk [6]. While AE that occurs soon after starting the ACEI treatment is common, many cases occur more than six weeks after initiating therapy. In addition, cases of late-onset angioedema are reported up to 23 years after the initiation of ACEI administration. Thus, AE can occur at any time during the treatment with an ACEI [4,7]. Recently it was estimated that the average duration of ACEI use among patients presenting with AE exceeds two years [8]. Such a time course represents a problem for the healthcare provider and the patient; for the clinician, the diagnosis may be difficult due to not recognizing the association, whereas for the misdiagnosed patient, the delay prolongs a potentially dangerous situation. ACEI-AE is more common (0.1–0.7% of treated patients) than other rare BK-mediated AE, such as hereditary angioedema (HAE), due to complement C1 esterase inhibitor (C1-INH) deficiency (1:10,000–1:50,000 prevalence), which is more common than acquired angioedema with C1 inhibitor deficiency (1:100,000 to 1:500,000 prevalence) [2,9,10].

## 3. Diagnostic Approach in Drug-Induced BK-Mediated AE

Currently, the diagnosis of drug-induced AE mediated by BK is clinical. There are no available laboratory tests to diagnose this non-allergic adverse reaction routinely. However, resolution following the discontinuation of the drug confirms the diagnosis.

ACEI-induced AE most often involves the head, neck, face, lips, tongue, and larynx. Rarely, it affects visceral organs. Life-threatening edema of the upper airway presents in about 25–40% of cases of ACEI-AE. ACEIs are the most common cause of BK-mediated AE, which can be life-threatening.

BK-mediated AE episodes induced by antihypertensive drugs acting on the renin–angiotensin–aldosterone system (RAAS), such as ACEIs, blockers of angiotensin II type 1 receptors (ARBs), and direct renin inhibitors (DRIs), primarily affect the head and neck region and may occur even after several years of uneventful treatment. Diagnosis is clinical but not easy for clinicians unfamiliar with such an adverse reaction, as the appearance resembles allergic AE. No specific diagnostic biomarkers are available, and no officially approved treatment is currently accessible [11].

Awareness of the epidemiology and risk factors for ACEI-AE is critical for appropriately managing any patient treated with ACEI, because it is associated with high morbidity (intubation, intensive care unit admissions, and hospitalizations) and fatal reactions have been documented [6]. BK-mediated AE can occur due to vasodilation and increased vascular permeability, mainly due to the inflammatory mediator BK. This form of AE can result from either overproduction of BK or, more frequently, from inhibition of BK degradation. Pruritus and urticaria are absent, mast cells being not involved, and it most commonly affects the lips, tongue, face, and upper airways. The intestine may also be involved, presenting as acute abdominal pain with diarrhea or other gastrointestinal symptoms, but this presentation may be less usually recognized. The reason why certain regions of the body are more often affected has yet to be comprehended [1,8].

## 4. Metabolism of BK and Its Receptors

The vasoactive nonapeptides BK and kallidin (Lys-BK) exert their bioactivities by binding to constitutively expressed kinin B2 receptors before being metabolized by multiple peptidases. In various cells and tissues, neutral endopeptidase (NEP, neprilysin) plays a significant role in the degradation of BK. In human plasma, BK is metabolized mostly by three metallopeptidases. Angiotensin I-converting enzyme (ACE) constitutes the main degradation pathway. Carboxypeptidase N (CPN) transforms BK and kallidin into active metabolites, des-arginine9-BK and Lys-des-Arg9-BK, respectively. This is a minor metabolic plasmatic pathway unless ACE is inhibited. Also, aminopeptidase P (X-pro aminopeptidase, proline aminopeptidase, APP) plays an important role in the metabolism of kinins in plasma, mostly for des-Arg9-BK. Although the carboxy-truncated metabolites of BK are largely inactive under normal conditions, but may be agonists of inflammation-regulated kinin B1 receptors [1,9,12]. By assessing the gene expression of metallopeptidases and kinin receptors in the oropharyngeal pig tissues, it was revealed that B2 receptor regulation might be modulated in a tissue-specific manner through ACEI treatment with mRNA upregulation in the tongue and laryngeal tissues [12].

In an overview of the renin–angiotensin–aldosterone system (RAAS), renin is secreted in the kidney by the juxtaglomerular cells in response to decreased renal perfusion pressures; the precursor angiotensinogen produced in the liver is converted by renin to produce the weak vasoconstrictor angiotensin I, which is further metabolized in the lungs by the ACE to create the potent vasoconstrictor angiotensin II (ATII), which acts on vascular endothelial receptors. The two types of ATII receptors, AT1 and AT2 receptors, which are present in the heart and vasculature smooth muscle, mediate its vasoconstrictive action. In addition, it also acts on the zona glomerulosa of the adrenal cortex to stimulate the release of aldosterone. This steroid hormone acts on the kidneys to promote sodium and water retention. Therefore, ACEI effects result from the inhibition of ACE, ultimately impacting the RAAS cascade and causing a decrease in systemic blood pressure [7,13].

Kinins are a group of active peptides released into body fluids and tissues following the enzymatic action of kallikreins on kininogens, which occurs through a complex proteolytic cascade of events called the kallikrein–kinin cascade. This contact activation pathway is initiated when factor XII (Hageman factor) binds to damaged tissue, becoming activated through conversion to factor XIIa. This converts prekallikrein to plasma kallikrein, and these two proteins autoactivate each other through a positive feedback loop. Plasma kallikrein cleaves high-molecular-weight kininogen (HMWK), producing BK. The binding of BK to its B2 receptors induces vasodilation and increased endothelial permeability, generating the characteristic manifestations of an acute episode of AE [2].

BK is generated through the cleavage of HMWK by activated kallikrein, which occurs through the contact system via factor XII, which can be activated by plasmin through the fibrinolysis pathway. In addition, the C1 esterase inhibitor (C1-INH), which acts as a brake on the complement system, blocks BK overproduction via the contact system [14,15].

ACE (also known as kininase II) plays a significant role in the renin–angiotensin–aldosterone system through two proteolytic mechanisms: conversion of angiotensin I to angiotensin II, and degradation of BK. These two actions make ACE inhibition a principal target in the treatment of hypertension, myocardial infarction (MI), heart failure, and type I diabetic nephropathy [2]. ACE is a crucial peptidase involved in the degradation of the vasoactive nonapeptide BK with a short half-life (about 17 s) to inactive metabolites. BK stimulates constitutively expressed B2 receptors, thus inducing vasodilation and vascular permeability and stimulating the release of SP from sensory fibers with further increased vascular permeability [16,17,18].

**Figure 1:**
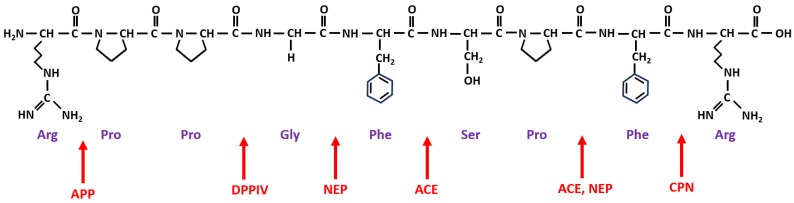
Structure of BK and sites of cleavage by significant enzymes related to drug-induced AE. Note: The amino acid sequence of BK is Arginine (Arg)–Proline (Pro)–Proline (Pro)–Glycine (Gly)–Phenylalanine (Phe)–Serine (Ser)–Proline (Pro)–Phenylalanine (Phe)–Arginine (Arg). Figure adapted from [23] for a simplified presentation of the sites of cleavage of BK by different important enzymes, marked with red arrows for ACE (angiotensin-converting enzyme), APP (aminopeptidase P), NEP (neutral endopeptidase), CPN (carboxypeptidase N), and DPPIV (dipeptidyl peptidase IV).

The amino acid sequence of BK is presented in Figure 1, along with the sites of cleavage by different enzymes significant in the pathogenesis of drug-induced BK-mediated AE. Table 1 presents such selected enzymes involved in BK metabolism, which are important for enhancing our understanding of its underlying mechanisms. By focusing on these enzymes, clinicians can better comprehend the complexities of BK-related drug-induced AE. Three specific metalloproteinases are involved in the primary degradation pathways of BK: ACE, APP, and NEP. Secondary enzymes include dipeptidyl peptidase-4 (DPP-IV) and CPN (kininase I).

ACEIs rapidly and strongly reduce ACE activity, but only weakly affect APP and NEP [13,19]. When ACE is inhibited by ACEIs, other BK metabolic enzymes, APP and NEP, along with DPP-4 and CPN, play a more significant role in degrading BK, des-Arg9-BK, and SP. These three mediators play critical roles in the pathophysiology of ACEI-AE, with BK having a central role in this BK-mediated AE. A secondary role is played by des-Arg9-BK, an active metabolite of BK formed primarily due to the CPN. The activities of the des-Arg9-BK, a selective agonist of the inducible B1 type receptors for kinins, are also short-lived because of its breakdown by APP, DPP-4, and ACE2. SP, inactivated primarily by the DPP-4 but also through NEP and ACE, also plays a secondary role. BK, des-Arg9-BK, and SP are challenging to measure due to their extremely short half-lives and the variability between serum and tissue concentrations in different tissues. Inhibition of ACE by ACEI impacts the degradation pathways of these compounds, and their elevated levels are associated with AE; however, multiple and redundant enzyme pathways may be involved [13,20,21,22].

**Table 1:** Important selected enzymes involved in BK metabolism related to drug-induced AE.

Enzyme Name and Role in BK Metabolism	Abbreviation		
Proteases involved in BK formation		type	location
plasma kallikrein	Pka	serine	plasma
tissue kallikreins	TK	serine	plasma, tissue
factor XIIa	αFXIIa	serine	plasma
plasmin	Pla	serine	plasma
Peptidases involved in BK degradation		alias	products
angiotensin-converting enzyme	ACE	kininase II	BK-(1-5), BK-(1-7)
carboxypeptidase N	CPN	kininase I	BK-(1-8) [des-Arg9-BK]
aminopeptidase P	APP	X-Pro aminopeptidase	BK-(2-9)
aminopeptidase N	APN	CD13	BK-(2-9)
neutral endopeptidase	NEP	neprilysin, CD10	BK-(1-7), BK-(1-4)
dipeptidyl peptidase IV	DPP-IV	CD26	BK-(4-9)

Note: BK = Bradykinin; Table adapted from [23] for a better understanding of BK-related drug-induced AE.

Defects or deficiencies in enzymes controlling BK catabolism, such as reduced APP activity, decreased DPP-4 antigen and activity levels, and slower degradation of des-Arg9-BK, predispose patients to develop AE when taking an ACEI [20,21,22]. Unlike patients with C1-INH deficiency, those with ACEI-AE show no evidence of the cleavage products of HMWK in their plasma, despite high plasma concentrations of BK. Because the cleavage of HMWK generates BK, the pathogenic mechanism of ACEI-AE resides in the catabolic part of BK metabolism [24]. The risks of ACEI-AE are not related to the dose or the specific agent administered (it being a class-adverse reaction) [25,26,27].

## 5. Drug-Induced BK-Mediated AE

ACEIs (drugs with the suffix ‘pril’) are classified as sulfhydryl-containing agents (captopril, zofenopril), dicarboxylate-containing agents (enalapril, ramipril, quinapril, perindopril, lisinopril, benazepril, imidapril, trandolapril, cilazapril), and phosphonate-containing agents (fosinopril). It is necessary to mention that sulfhydryl-containing ACEIs may be involved in adverse events such as skin eruptions [28]. No data suggest significant differences between ACEIs regarding AE risk [6].

The most consistent epidemiological factor associated with increased risk of ACEI-AE is prior AE during ACEI treatment, with a ten-fold increase in AE recurrence in patients continuing ACEI administration compared with those who stopped it [25].

A female predominance has been reported, with a high proportion of those experiencing an increase in acute episodes during menstrual periods, pregnancy, oral contraceptives, and hormone replacement therapy. Estrogen is one of the determinants in regulating low-molecular-weight kininogen gene expression in vivo. High levels of estrogens, such as during oral contraceptive use, are reported to reduce plasma C1-INH levels, increase transcription of FXII protein, and increase plasma prekallikrein levels, favoring an increase in bradykinin production. Estrogens may also increase the expression of bradykinin type 2 receptors [28,29,30].

Some additional BK-related risk factors for ACEI-AE are predisposing genetic traits. For example, the incidence of ACEI-AE is up to five times higher in people of African descent, a possible explanation being the presence of polymorphisms in genes encoding enzymes APP (XPNPEP2, location Xq26.1) and NEP (MME, location 3q25.2), which occur at a higher rate in African Americans. Such polymorphisms are associated with lower levels of circulating enzymes responsible for the degradation of BK and its active metabolite, des-Arg9-BK [20,21,31,32,33]. In other populations, genetic polymorphisms in the XPNPEP2 gene that encodes APP reduce plasma APP activity with higher levels of BK and des-Arg9-BK, and are associated with a higher occurrence of ACEI-AE [34,35].

Moreover, an association between reduced plasma APP activity and anaphylactoid reactions during hemodialysis was reported in patients treated with ACEIs [36]. Finally, polymorphism of the ACE gene (location 17q23.3) does not affect the ACEI-AE [37]. Furthermore, in a genome-wide association study, no significant associations of any single-nucleotide polymorphism (SNP) with AE were found in either African Americans or those of European ancestry [38].

Other potential BK-related risk factors may include using non-selective nonsteroidal anti-inflammatory drugs (NSAIDs) and a history of angioedema related to NSAIDs. Because some of the effects of BK on B2R are mediated by nitric oxide (NO) and PGI2, acetylsalicylic acid and other NSAIDs can exacerbate BK-mediated forms of AE. Still, there is no clear rationale to avoid NSAIDs further unless the associated episodes of AE continue to occur after approximately six months of ACEI discontinuation [6,39,40,41,42].

The concomitant use of ACEIs and NSAIDs may be avoided in patients with a suggestive history of AE, as the combination leads to more severe episodes [28]. Underlying hereditary or acquired C1 inhibitor deficiency or dysfunction predisposes individuals to episodes of BK-mediated AE, with some patients being asymptomatic until exposed to ACEI [43,44,45]. Selected patients diagnosed with ACEI-AE should be tested for hereditary angioedema, as the AE attacks after initiating treatment with an ACEI may unmask a previously asymptomatic disease [46,47]. Obtaining a blood sample for determining the serum level of complement C4 is reasonable if the patient has a family history of AE or has an underlying lymphoproliferative disorder (such as lymphoma or monoclonal gammopathy of uncertain significance) or other malignancy [47,48,49]. A history of ACEI-associated cough was reported as a risk factor for cough and AE, involving increases in BK and/or SP [44,50]. Even without AE, ACEI as a cause of cough should be considered even after years of uneventful therapy [6].

Treatment with a mammalian target of rapamycin inhibitors (mTORIs), such as sirolimus (rapamycin) or everolimus, in kidney transplant patients, predisposes individuals to AE when administered with ACEIs (showing a 6.6% incidence in Caucasian patients with combination therapy), due to impaired endothelium-dependent relaxation in response to BK, suggesting that mTORIs interfere with the BK pathway [51,52,53,54].

In addition, cardiac and renal transplant patients are at increased risk of ACEI-AE, possibly due to cyclosporine and other immunosuppressants’ effects on DPP-IV activity [13,55,56]. By contrast, a lower risk of ACEI-AE in diabetic patients untreated with gliptins may be explained by increased DPP-IV activity associated with hyperglycemia [13,57,58]. But diabetes and obesity have instead been shown to increase the severity of AE events [59]. In patients with non-histamine-mediated AE, ACEIs should be discontinued immediately, with the prescription of an alternative class of medication with a different mechanism of action. ACEI-AE resolves within 24 to 72 h (usually ≤48 h) once the drug is discontinued. However, if ACEI treatment is continued, there is an increased and unpredictable rate of AE recurrence, and episodes may become more severe or life-threatening. Patients should be informed that after cessation of the ACEI, about half of the patients may have recurrences of AE, usually occurring within the first month after the ACE inhibitor is discontinued. However, recurrences may continue for six months in some patients. Therefore, even if the cause of a patient’s AE is considered unclear, or in cases of a personal history of idiopathic AE, it is still advisable to discontinue ACEIs [4,9,60,61].

Rapid progression of symptoms within the first six hours of AE onset, anterior tongue swelling, vocal changes, drooling, and dyspnea are associated with intubation for ACEI-AE [60].

The standard of care for ACEI-AE is non-specific, and consists of discontinuing the ACEI, airway monitoring, and acute airway management if needed. Systemic H1 antihistamines, corticosteroids, and adrenaline are often administered but are ineffective in this non-allergic form of AE. Drugs proven efficient in acute episodes of hereditary AE, another type of BK-mediated AE, have been studied in ACEI-AE. However, ACEI-AE is caused by the persistence of BK due to a deficiency in metabolism rather than overproduction, so it is only partially intuitive that these drugs would be effective. Moreover, unopposed effects of SP or other peptide substrates of ACE may contribute to AE, even when the B2 receptor is blocked [8,62,63].

Several case reports and case series suggested that plasma-derived C1 INH [64,65,66,67] and fresh frozen plasma (FFP) may be effective in treating ACEI-AE, and that this may prevent the need for invasive interventions in many patients [15,68].

Details of a clinical trial of C1-INH for the treatment of ACEI-AE were recently published [69]. No differences in discharge time reached statistical significance in two small randomized controlled studies in patients with ACEI-AE treated with ecallantide, a synthetic kallikrein inhibitor [70,71].

Tranexamic acid is an antifibrinolytic agent that reduces BK production through its blockade of converting plasminogen to plasmin and, subsequently, pre-kallikrein to kallikrein. It has been utilized in ACEI-AE in small case reports and series, suggesting it may be a safe and effective treatment option [72,73].

Studies assessing the use of icatibant, a synthetic bradykinin B2 receptor antagonist, in the emergency department for ACEI-AE need to be more consistent. Case reports, small case series, and one randomized trial initially supported using icatibant for ACEI-AE, with all patients showing clinical improvement and a symptom regression time of 0.5–7 h following icatibant therapy [74,75,76,77]. However, these were followed by two more extensive randomized controlled studies that reported no difference in time to discharge or time to symptom resolution between icatibant and placebo [8,78]. An explanation for this may be the longer time interval from the onset of angioedema to icatibant treatment. Moreover, it was suspected that the effect of icatibant would differ between Europeans of Caucasian descent and patients of African descent, or between men and women, but this was not confirmed in post-hoc analyses [8].

Therefore, further investigation is required before establishing the effectiveness of icatibant for drug-induced AE in the emergency department [15].

Combinations of neprilysin inhibitors were developed for heart failure patients to increase vasodilatory natriuretic peptides and prevent counterregulatory activation of the angiotensin system. Neprilysin or neutral endopeptidase (NEP) cleaves various mediators such as natriuretic peptides, adrenomedullin, angiotensin I and II, endothelin, and BK and SP. Omapatrilat, a vasopeptidase inhibitor with combined neprilysin and ACE inhibition, had a three times higher risk of AE compared with enalapril alone, and was therefore not considered for approval in clinical practice. The combination sacubitril–valsartan, an angiotensin receptor–neprilysin inhibitor combination drug (ARNI), was instead approved for managing heart failure with reduced ejection fraction. An increased risk of AE among sacubitril–valsartan users who recently switched from ACEI or ARB compared with sacubitril–valsartan new users was observed. However, a known history of AE related to previous ACEI or ARB therapy represents a contraindication, as does hereditary or idiopathic AE [79,80].

ARBs, also known as angiotensin II receptor antagonists, selectively block angiotensin II type 1 (AT1) receptors and do not inhibit the catabolism of BK; AE was not conceptually anticipated as an adverse reaction. However, AE was associated with ARB treatment, such as losartan, valsartan, candesartan, olmesartan, irbesartan or telmisartan; that said, the risk appears to be considerably lower compared with ACEIs [81,82,83,84,85].

The pathophysiology of ARB-induced AE is still uncertain. Research in human subjects suggests that ARBs increase BK levels via indirect mechanisms. ARBs prevent angiotensin II from binding the angiotensin type 1 receptor, resulting in elevated angiotensin II levels. Elevated angiotensin II may stimulate angiotensin type II (AT2) receptors, which generally reduce ACE activity. Stimulation of AT2 receptors, therefore, may reduce ACE activity and increase BK levels. In addition, an upregulation of angiotensin type 2 receptors may appear in an increased level of angiotensin II. Moreover, losartan acts as a partial agonist of the B2R through direct binding and activation, suggesting the B2R agonism of some ARBs [86,87,88]. However, earlier systematic reviews described a 1.5% to 10% rate of recurrent AE in patients with a history of ACEI-AE who were switched to an ARB [89,90]. Subsequent studies have not found ARBs to be associated with higher rates of AE than other antihypertensives such as beta-blockers [91,92,93]. For patients with a life-threatening AE episode, both ACEIs and ARBs should be avoided. For patients with less severe ACEI-AE, ARBs may be used with close monitoring in patients with conditions that may particularly benefit from RAAS inhibition, such as chronic heart failure [4]. ARBs should not be avoided in all patients with ACEI-AE if ARB therapy offers an advantage over other antihypertensives for an individual patient. When switching from an ACEI to an ARB, the patient must understand the risk of recurrence of AE due to ACEI. A more careful approach is to wait about one to two months after an ACEI is discontinued before starting an ARB, if the patient can safely be treated with such an approach. Only a tiny percentage of patients with ACEI-AE continue to present this manifestation when switched to an ARB [94].

A direct renin inhibitor (DRI) such as aliskiren should not confer a risk of BK-induced AE, because the mechanism of action is conceptually different from that of ACEIs or ARBs. Aliskiren may increase tissular BK levels independent of renin inhibition or changes in BK metabolism, but may be associated with increased tissue kallikrein gene expression. AE has been reported with aliskiren, with the incidence of AE being reported even at similar rates to those with ACEIs. AE may appear at any time during treatment, and has occurred in patients with and without a history of AE with ACEIs or ARBs [93,95,96,97].

AE associated with thrombolytic agents was reported with intravenous recombinant tissue plasminogen activators (rt-PAs), such as alteplase or tenecteplase, used to treat acute ischemic stroke, and more rarely with streptokinase used for acute myocardial infarction. The incidence of this adverse reaction is about 2–8% in patients treated with thrombolytic therapy for acute ischemic stroke [98,99,100]. However, the risk is six times higher for patients treated simultaneously with ACEIs, and three times higher in women. The AE associated with such fibrinolytic agents is likely BK-mediated, as plasmin activates the coagulation factor XII of the contact system. The recombinant serine proteases cleave plasminogen to plasmin, and in addition to their thrombolytic effects, activate the kinin pathway by converting Factor XII to Factor XIIa, with increased production of BK and AE usually appearing a few hours after thrombolytic treatment. AE is asymmetric in many cases, and mainly contralateral to the ischemic hemisphere, suggesting that infarction of the insular cortex could lead to contralateral autonomic dysfunction [101,102,103]. In addition, cerebral damage secondary to brain ischemia can lead to BK release [104].

DPP-IV inhibitors, also known as gliptins (sitagliptin, vildagliptin, saxagliptin, linagliptin), are oral antidiabetic agents. The incidence and prevalence of DPP-4 inhibitor-associated AE are not known. However, healthcare providers should be aware that BK-mediated AE has been associated with gliptins, which inhibit the degradation of BK and SP, and that AE risk is higher when used together with ACEIs or immunosuppressive medications [55,87,105,106].

Statins (HMG CoA reductase inhibitors) may rarely induce BK-mediated AE in patients not receiving other drugs with a mentioned risk of AE. These drugs increase the number of B2 receptors on endothelial cells. Moreover, the effect of BK on its receptors may be amplified by statins. By increasing the release of NO and prostacyclin, these mechanisms might enhance a patient’s susceptibility to developing AE in the presence of circulating BK. The combination of increased B2 receptors caused by statins and activation of the kinin system caused by a calcium blocker such as amlodipine may hasten the onset of AE [107,108].

**Figure 2:**
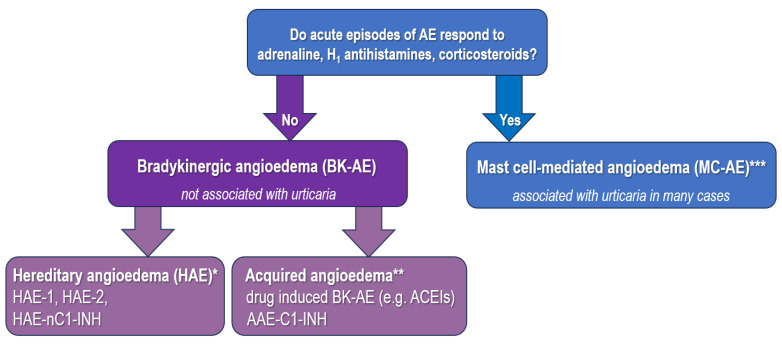
Overall presentation of different causes of angioedema classified by pathophysiology mechanisms, focusing on bradikinergic or BK-mediated angioedema (BK-AE) and drug-induced forms [3,47,109]. * Hereditary angioedema (HAE) with C1-INH deficiency with low antigenic and functional C1-INH levels (HAE-1, 85% of cases), HAE due to C1-INH dysfunction (HAE-2, 10% of cases) characterized by normal or elevated antigenic but low functional C1-INH levels, and HAE with normal C1-INH (HAE-nC1-INH, 5% of cases) due to various gene mutations. ** Acquired forms of BK-AE include drug-induced AE, such as AE induced by angiotensin-converting enzyme inhibitors (ACEIs), AE due to acquired C1-INH deficiency (AAE-C1-INH), and acquired idiopathic non-histaminergic AE. *** Mast cell-mediated angioedema (MC-AE) includes allergic IgE-mediated reactions (such as AE with anaphylaxis), nonallergic non-IgE-mediated reactions (such as AE due to non-steroidal anti-inflammatory drugs or infections), AE in chronic inducible urticaria or chronic spontaneous urticaria (sometimes referred to as idiopathic histaminergic acquired AE). The diversity of mediators involved in MC-AE is reflected also by the fact that patients with chronic spontaneous urticaria may respond to treatment with omalizumab but not to H_1_ antihistamines.

Finally, we underline that unlike histamine-mediated AE, BK-AE is not associated with pruritus and urticaria, and does not respond to adrenaline, H_1_ antihistamines, or glucocorticoids (Figure 2). The clinical characteristics of the most important forms of BK-AE are presented in Table 2.

**Figure 3:**
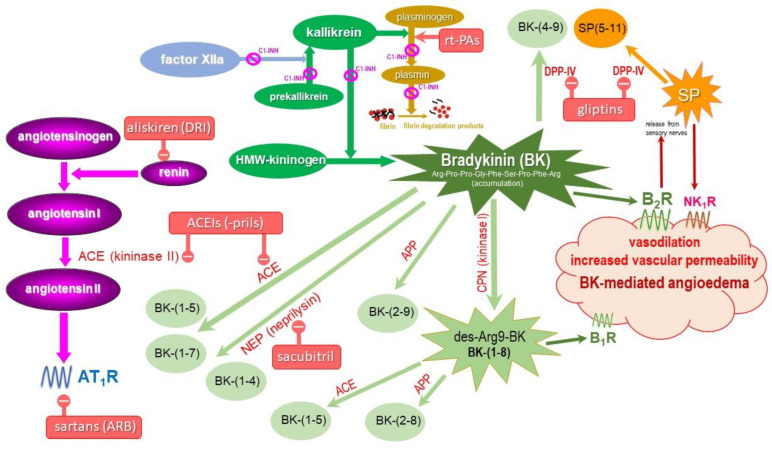
Metabolism of bradykinin (BK) and angioedema induced by cardiovascular and antidiabetic drugs: angiotesin-converting enzyme inhibitors (ACEIs), angiotensin receptor blockers (sartans), neprilysin inhibitors, direct renin inhibitor (aliskiren), gliptins (DPP-IV inhibitors), and recombinant tissue plasminogen activators (rt-PAs), figure created and adapted after [23,57,87,111].

Moreover, BK-mediated drug-induced AE does not involve drug-specific IgE antibodies, and there are no point-of-care or laboratory-based in vitro tests available to provide immediate treatment guidance. However, serum levels of complement C4 and tryptase may be considered to assist in diagnosing hereditary angioedema and angioedema associated with anaphylaxis, respectively. Supplemental oxygen, nasal trumpets, and bag-valve-mask ventilation may be helpful, but are not a substitute for intubation or a more aggressive approach such as cricothyrotomy, if needed. The allergy treatment for histamine-mediated AE is generally not effective for BK-mediated AE, therefore such a type of drug-induced AE, especially ACEI-AE, is treated by emergency medicine specialists in the Emergency Departments (ED) and their observation units, and, in case of selected patients with lingual AE and laryngeal AE, in an intensive care unit (ICU), because prompt airway intervention defined as intubation, cricothyrotomy, or tracheotomy, may be required. Nevertheless, standard pharmacological treatment is not contraindicated, and if the putative cause of AE is unknown, adrenaline, followed by H_1_ antagonists and corticosteroids, is administered. Fresh frozen plasma, C1-INH protein products, the kallikrein inhibitor ecallantide, and the bradykinin 2-receptor antagonist icatibant were considered for an acute therapeutic approach, similar to hereditary angioedema attacks; however, their efficacy is not conclusive in drug-induced BK-mediated AE due to its complex pathophysiology (Figure 3) involving the metabolism of BK, BK itself, and its B2 receptors inducing vasodilation and increased vascular permeability. B2 receptor-stimulated release of SP from the sensory nerves also increases vascular permeability, and even BK-related endogenous peptide fragments with biological activities (some mediated by the B1 receptors, such as BK-(1-8) or des-Arg9-BK, and some not mediated by the B1 or B2 receptors, such as BK-(1-7) and BK-(1-5) [2,8,57,110,111]).

## 6. Conclusions

Drug-induced AE is caused by increased production or decreased breakdown of vasoactive peptides, mainly BK. Many cardiovascular and antidiabetic medications may cause BK-mediated AE. For example, ACEIs and neprilysin inhibitors impede BK catabolism. ARBs may indirectly increase BK levels by increasing angiotensin II, which may decrease ACE activity. DPP-IV inhibitors reduce the breakdown of SP and BK. Moreover, thrombolytic agents and statins may also induce BK-mediated AE.

## Figures and Tables

**Table 2:** Clinical characteristics of the most important forms of BK-AE.

Feature	ACEI-AE	HAE
age of onset	adult	usually 2–13 years
location of AE attacks	lips, tongue, face	facial, laryngeal, peripheral, abdominal area
speed of AE attack onset	gradual, over few hours	gradual, over few hours rapid onset, possibly <1 h
duration of AE attacks	usually 24–48 h after drug discontinuation	3–5 days without treatment
association with urticaria	no	no
abdominal pain presentation	yes	usually not

Note: ACEI-AE = AE induced by angiotensin-converting enzyme inhibitors; HAE = hereditary angioedema. Table modified from [110] for a simplified, rapid clinical approach for clinicians.

## Data Availability

Not applicable.
